# MARITAL STATUS AND CHANGES IN FINANCIAL SATISFACTION DURING THE RETIREMENT TRANSITION: SOUTH KOREA AND THE US

**DOI:** 10.1093/geroni/igae098.1668

**Published:** 2024-12-31

**Authors:** Jeein Jang, Jeffrey Stokes, Jeffrey Burr

**Affiliations:** University of Massachusetts Boston, Boston, Massachusetts, United States; University of Massachusetts Boston, Boston, Massachusetts, United States; University of Massachusetts Boston, Boston, Massachusetts, United States

## Abstract

This study examines the impact of the transition to full retirement on the long-term financial satisfaction trajectories in two countries with different socioeconomic and health settings, and whether this relationship differs by marital status. Two harmonized, nationally representative cohorts of adults aged 51 and over are used: the Korean Longitudinal Study of Aging for South Korea and the Health and Retirement Study for the United States, with sample sizes of 31,900 and 33,356 individuals, respectively, followed up for a period of 11 years. The outcome is financial satisfaction measured as a composite score, and the predictor is the transition from full-time work to becoming fully retired (relative to remaining in full-time work). Linear Mixed-effect models show that retirement is associated with lower financial satisfaction at baseline and over time in both countries. Men have higher financial satisfaction than women in both studies. The magnitude of the effect of retirement on financial satisfaction is greater in South Korea than in the United States. This relationship is buffered by marital status in the United States, but no interaction term reaches statistical significance in South Korea. These findings suggest that cultural, social, or economic factors may influence the financial well-being of individuals during the transition to full retirement, given cross-national differences.

